# Anatomy and Physiology of Water Buffalo Mammary Glands: An Anatomofunctional Comparison with Dairy Cattle

**DOI:** 10.3390/ani14071066

**Published:** 2024-03-30

**Authors:** Daniel Mota-Rojas, Fabio Napolitano, Alfonso Chay-Canul, Marcelo Ghezzi, Ada Braghieri, Adriana Domínguez-Oliva, Andrea Bragaglio, Adolfo Álvarez-Macías, Adriana Olmos-Hernández, Giuseppe De Rosa, Ricardo García-Herrera, Pamela Lendez, Corrado Pacelli, Aldo Bertoni, Vittoria Lucia Barile

**Affiliations:** 1 Neurophysiology, Behavior and Animal Welfare Assessment, DPAA, Universidad Autónoma Metropolitana (UAM), Mexico City 04960, Mexico; 2 Scuola di Scienze Agrarie, Forestali, Alimentari ed Ambientali, Università degli Studi della Basilicata, 85100 Potenza, Italy; 3 División Académica de Ciencias Agropecuarias, Universidad Juárez Autónoma de Tabasco, Villahermosa 86040, Mexico; 4 Anatomy Area, Faculty of Veterinary Sciences (FCV), Universidad Nacional del Centro de la Provincia de Buenos Aires (UNCPBA), University Campus, Tandil 7000, Argentina; 5 Research Centre for Engineering and Food Processing, Council for Agricultural Research and Agricultural Economy Analysis (CREA), Via Milano 43, 24047 Treviglio, Italy; 6 Division of Biotechnology—Bioterio and Experimental Surgery, Instituto Nacional de Rehabilitación-Luis Guillermo Ibarra Ibarra (INR-LGII), Mexico City 14389, Mexico; 7 Department of Agricultural Sciences, University of Naples Federico II, 80055 Portici, Italy; 8 Faculty of Veterinary Sciences (FCV), Universidad Nacional del Centro de la Provincia de Buenos Aires, CIVETAN, UNCPBA-CICPBA-CONICET (UNCPBA), University Campus, Tandil 7000, Argentina; 9 Research Centre for Animal Production and Aquaculture, Consiglio per la Ricerca in Agricoltura e l’Analisi dell’Economia Agraria (CREA), Via Salaria 31, 00015 Monterotondo, Italy

**Keywords:** udder morphology, *Bubalus bubalis*, prestimulation

## Abstract

**Simple Summary:**

Significant differences in milk yield are observed between water buffalo (*Bubalus bubalis*) and dairy cattle (*Bos taurus*). Since these differences could be related to the anatomofunctional characteristics of the buffalo and dairy cattle udder, the present review aims to analyze the anatomical and physiological characteristics of the mammary glands and udders of water buffalo by making an anatomofunctional comparison with dairy cattle. It will also discuss the knowledge generated around the physiological regulation of milk ejection in the water buffalo. According to the revised literature, the buffalo’s udder and teat measurements are smaller than dairy cattle, having a narrower teat canal due to a thicker sphincter muscle. These elements and the negligible amount of milk stored in the cisternal fraction of water buffalo influence milk yield and the requirement for prestimulation and external elements to promote milk ejection in the species.

**Abstract:**

The present review aims to analyze the anatomical and physiological characteristics of the mammary gland and udders of water buffalo by making an anatomofunctional comparison with dairy cattle. It will also discuss the knowledge generated around the physiological regulation of milk ejection in the water buffalo. It was found that buffalo’s average udder depth and width is approximately 20 cm smaller than *Bos* cattle. One of the main differences with dairy cattle is a longer teat canal length (around 8.25–11.56 cm), which highly influences buffalo milking. In this sense, a narrower teat canal (2.71 ± 0.10 cm) and thicker sphincter muscle are associated with needing higher vacuum levels when using machine milking in buffalo. Moreover, the predominant alveolar fraction of water buffalo storing 90–95% of the entire milk production is another element that can be related to the lower milk yields in buffalo (when compared to *Bos* cattle) and the requirements for prolonged prestimulation in this species. Considering the anatomical characteristics of water buffalo’s udder could help improve bubaline dairy systems.

## 1. Introduction

Water buffalo (*Bubalus bubalis*) is one of the main species farmed for dairy purposes, mostly in Asia (97% of the buffalo population) [1,2,3]. Buffalo can be considered the second most important dairy animal, after *Bos* cattle, producing approximately 73.2 million tons of milk annually, with an annual growth rate of 3.1% [4,5]. Lactation milk yield in water buffalo ranges between 1500–1800 kg [6] and can reach up to 1983 kg in Murrah or 2542.60 kg in Jafarbadi buffalo [7]. However, these values are below the average milk yield of *Bos* cattle (between 9000 to 11,000 kg in Holstein-Friesian) [8]. In comparison with cow milk, it is known that buffalo milk has higher values for fat (41 vs. 70 g/kg, respectively), lactose (48.0 vs. 52.1 g/kg, respectively), and protein (57.8 vs. 58.2%, respectively) [9]. Moreover, buffalo milk is creamier and due to its high nutritional value is used to produce dairy subproducts such as butter, butter oil, cheese (particularly mozzarella cheese with the milk of Italian Mediterranean buffalo), condensed and evaporated milk, ice cream, and yogurt [9,10]. One reason that could explain the differences in milk performance could be the anatomical differences between the mammary glands (MG) of water buffalo and *Bos* cattle.

The (MG) is a specialized milk synthesis organ in mammals [11,12], considered histologically as a modified alveolar lobe-type sweat gland, evolved for milk production [11,13,14]. In ruminants, the MG is the udder. It is made up of two or four mammary complexes, each consisting of the MG and the teats, and is normally located bilaterally, symmetrical, and parallel to the midline on the ventral wall of the trunk [15,16]. The MG has a continuous secretion during lactation, and the milk is stored in the lumen of the secretory alveoli and the ductal system of the gland until its elimination, either by suckling the offspring or by milking [13,17,18,19].

The size, shape, structure, composition, and activity of the udder differ according to the developmental stage of the animal (e.g., embryonic, prepubertal, pubertal, pregnancy, lactation, involution) and to the species [12,13,20,21,22,23,24,25]. It is known that buffalo’s udder is smaller than dairy cattle. The buffalo’s udder depth (10.8 ± 1.6 cm), width (29.1 ± 4.1 cm), and length (64.2 ± 7.3 cm) (in Nili-Ravi animals) are smaller than Holstein cows (30.63, 50.52, and 50.78 cm, respectively) [26]. Moreover, buffalo have a narrower teat canal (2.71 ± 0.10 cm) [27] and a thick sphincter muscle [28]. The mammary venous system is also different in buffalo, having larger branches from the two major longitudinal veins more blood vessels and nerve fibers, and a smaller sphincter opening [29,30]. These characteristics can have a significant effect on an animal’s milk yield, as seen in *Bos* cattle [31], and can also be associated with milking difficulties [12], or udder health due to the particular morphology of the teat [24]. Therefore, this study aims to analyze the anatomical and morphological characteristics of the MG, addressing the main anatomofunctional differences in comparison to dairy cattle (*Bos taurus*), possibly influencing the storage capacity of the udder. It will also discuss the knowledge generated around the physiological regulation of milk ejection in water buffalo.

## 2. Anatomy and Structure of the Mammary Gland in Water Buffalo and Dairy Cows

### 2.1. Localization and Anatomical Characteristics

In buffalo, as well as in dairy bovine, the udder is located in the inguinal area [32]. Its shape is saccular, rounded, and transversely flattened, and the base is slightly concave and inclined obliquely towards the ventral side of the animals [33]. The udder is covered externally by soft and elastic skin, provided with fine hair except on the teats, which have wrinkles, and an epidermis rich in pigmented cells, which protects against solar radiation [23].

The udder of buffalo and cows is divided into four parts (or quarters), with two mammary quarters, right and left, in the cranial side and two in the caudal side [34], each consisting of an independent functional unit with glandular body or mammary body and a teats. The median intermammary groove produced by the tension of the median suspensory ligament divides the udder into right and left glands, while the lateral-left and right-suspensory ligaments provide support to the udder [16]. Nonetheless, the suspensory ligament of water buffalo is less developed than cattle [21]. Although there is no specific data on water buffalo, the weight of the udder is approximately 5 to 10% of the animal’s live weight, which is affected by various factors, such as the age of the animal, the number of lactations, the amount of milk present in the gland, and genetic heritage [35].

**Figure 1:**
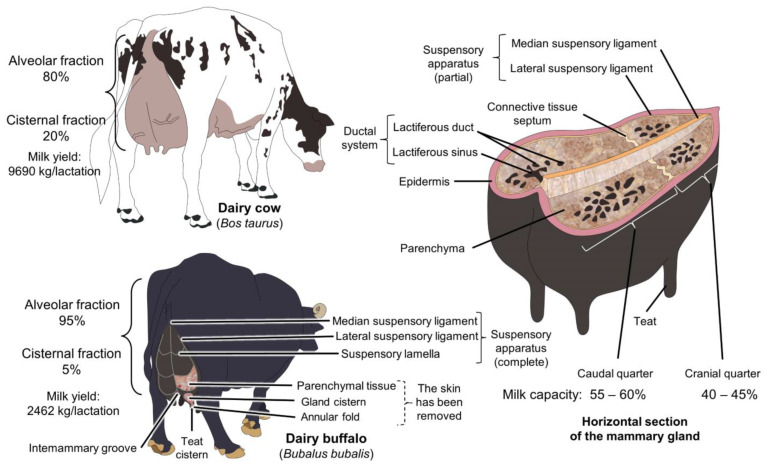
Morphologic comparison of the udder of dairy cattle and dairy buffalo. The main difference is the greater development of the udder in cattle resulting in higher milk yields.

Several studies have questioned the theory of independent mammary gland quarters, showing changes in adjacent healthy quarters when one quarter has mastitis [36,37,38]. The caudal (posterior) pair of MG is slightly more developed, contains 25 to 50% more secretory tissue, and ejects a higher percentage of milk than the cranial (anterior) pair (55–60% and 40–45%, respectively), [39]. In buffalo, the caudal quarters are slightly more developed than the cranial quarters, with a higher percentage of secretory tissue (25 to 50%, respectively), which can produce more than 50% of the total milk secreted (Figure 1) [40]. Moreover, the caudal quarters are 1.5 cm larger than the cranial ones [41]. In this sense, it has been found that caudal quarters have an average length of 3.7 ± 0.2 cm, while the cranial ones have 3.0 ± 0.1 cm [42].

### 2.2. Morphologic Characteristics

Udder morphology highly influences the productivity and milk composition in ruminants. During the evolutionary period of embryogenesis and sexual maturity, the MG alters its shape with successive lactations, and it has been mentioned that the average conformation of the MG in buffalo stabilizes in the fifth lactation [43,44,45].

In buffalo, characteristics of the udder such as depth, length, and width are associated with milk yield due to their direct relationship with mammary volume. In cattle (*Bos taurus* and *Bos indicus*) voluminous udder have a greater amount of secretory tissue and, consequently, a greater milk yield [46]. Likewise, the increase in the length of the udder influences the storage capacity of the gland [41,47].

Among the anatomical differences between buffalo and dairy cows, it has been reported that the latter has a greater development in the mammary complex, which has allowed them to synthesize large quantities of milk [12,29]. In *Bos* cattle, approximately 70–80% of milk is stored in the alveolar fraction (alveoli and small milk ducts), while the cisternal fraction (large mammary ducts and cisternal cavity) contains 20–30% [48,49,50]. In contrast, the alveolar fraction of water buffalo stores 90–95% of the entire milk production [47,51], and some authors state that no milk is stored in the cisternal fraction, or that it only represents 4.9 ± 0.1% [52]. This is one of the reasons why milk letdown requires longer periods for buffalo and udder stimulation and oxytocin (OXT) administration is critical for dairy buffalo to make the milk from the alveolar fraction available [53]. This characteristic, caused by incomplete milk extraction, makes buffalo susceptible to immediate loss of milk production and apoptosis in the mammary epithelium [31].

**Figure 2:**
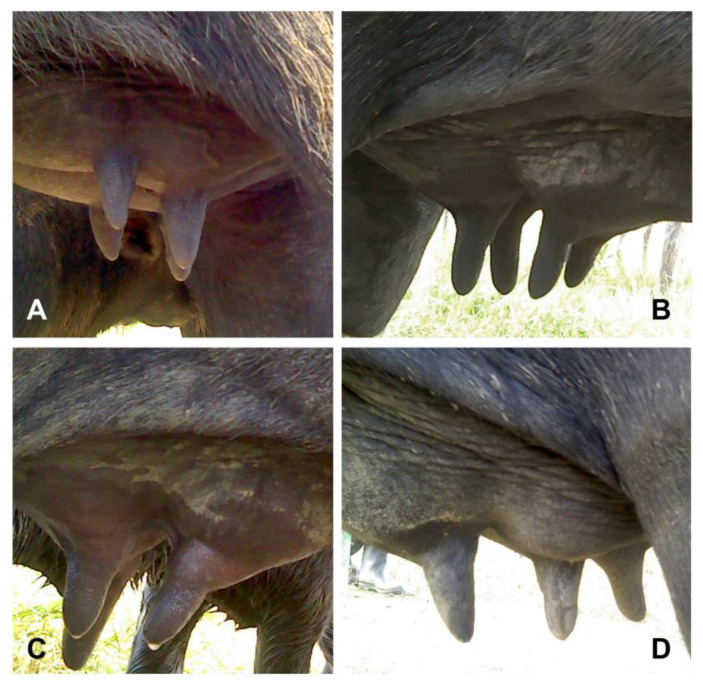
Examples of different udder and teat shapes in water buffalo. Shapes of the udder and nipples, respectively, have been described as (**A**), divided and conical; (**B**), pendulous and cylindrical; (**C**), goat shaped and bottle shaped; (**D**), pear shaped and pear shaped.

The shape of the udder and teats also greatly differs in buffalo. In bubaline females of the Murrah breed and their crossbreeds, the predominant anatomical shapes of the udder have been described as bowl, globular, goaty, and pendulous udder, representing the 61, 17, 9, and 13% of shape in Murrah buffalo [54]. Likewise, five anatomical shapes of teats have been described, where the most common is cylindrical (52.5%), followed by pear shaped (18%), bottle shaped (11.0), conical (10.5%), and funnel (8.0%) [54] (Figure 2). Particularly in buffalo, longer and thicker teats with narrower channels are predominant [55]. In addition, a more closed sphincter, a typical anatomical characteristic of water buffalo, is considered an advantage of the species against mastitis [12]. However, other studies have shown a similar frequency of mastitis between dairy cattle and water buffalo [56]

In this sense, in different breeds of buffalo, the morphometric traits have been evaluated, showing that the Mediterranean buffalo breed has teats with a length of 6.3 to 8.5 cm, while in the Murrah breed the teats of the cranial quarters have a length between 5 and 14 cm and the caudal quarters between 8 and 16 cm [12]. In these same breeds, the percentage of cisternal fractions also differs, recording 7.8% in Mediterranean buffalo [57] and 4.9% in Murrah females [52]. Thomas et al. [52] reported that Murrah buffalo had an average gland cisternal area of 69.6 ± 4.6 cm^2^, 51.61 ± 4.8 cm^2^, and 26.01 ± 4.8 cm^2^ during early, mid, and late lactation, showing that even within the same animal the morphology of the udder differs according to the reproductive stage.

The teats tip shapes are grouped within five categories: pointed, rounded, flat, disc or plate shaped, and inverted [58], and they are considered part of the passive defense mechanism against the invasion of microorganisms into the udder [59]. Prasad and Laxmi [60] have even reported that the shape of the udder might be related to the animal’s temperament. The authors found that the majority of Murrah buffalo with all udder shapes and with conical, pear, cylindrical, and funnel-shaped teats were of docile temperament. In contrast, restlessness was more common in buffalo with bottle-shaped teats. However, there was no significant variation in the frequencies of buffalo with different temperament scores among various udder and teat shape categories. The average daily milk production in the categories docile, slightly restless, restless, aggressive, and nervous categories was 6.70 ± 0.15, 6.50 ± 0.34, 5.70 ± 0.26, 4.90 ± 0.30, and 4.60 ± 0.34 kg, respectively.

A study made by Boselli et al. [27] was carried out on Mediterranean Italian buffalo to determine the teat length, diameter, thickness, and teat cisternal diameter. The authors found that teat length (8.33 ± 0.22 cm vs. 7.01 ± 0.17 cm), diameter (3.41 ± 0.06 cm vs. 3.19 ± 0.08 cm), and teat cisternal diameter (0.91 ± 0.03 cm vs. 0.81 ± 0.03 cm) were higher in the hind quarters than in the fore quarters and that these parameters were correlated with higher milk flows (r = 0.27). The mean teat canal length (2.78 ± 0.11 cm) found by Boselli et al. [27] was lower than that reported in Brown Swiss x German Braunvieh cows (5.6–6.7 cm) [61], which is in contrast to what was reported in Murrah animals, in which an average teat canal length for fore and hind teats was 8.25–11.56 cm and 10.71–14.31 cm, respectively [52]. Considering the length of the buffalo’s teat canal is relevant during milking because buffalo require higher vacuum levels when machine milked [27,52].

Teat anatomy has also been shown to affect udder preparation in Mediterranean buffalo [44]. In this sense, Ambord et al. [44] evaluated teat wall thickness, diameter, teat canal length, and teat cisternal diameter before and after a 3 min prestimulation with a vacuum teat cup. Differences were found before and after this period for teat canal length (23.6 ± 1.1 mm vs. 14.8 ± 0.7 mm), teat canal diameter (29.2 ± 0.5 mm vs. 29.6 ± 0.6 mm), teat cistern (3.9 ± 1.0 mm vs. 8.9 ± 0.9 mm), and teat canal wall (12.6 ± 0.5 vs. 10.3 ± 0.4 mm). They also found that teat cistern length, thickness, and teat cisternal diameter correlated with the vacuum needed to open the teat canal (r = 0.82) [57]. These findings emphasize the importance of taking into account anatomical variations in buffalo to improve milking practices. Table 1 summarizes the main morphologic characteristics of the udder and teats in different breeds of water buffalo.

### 2.3. Internal Structure

In general, the macroscopic anatomy of the udder differs between ruminants, since the number of glands is different according to the species [12]. The MG is formed by a branched network of ducts that end in the alveoli [11]. It undergoes cyclical developmental changes during gestation, lactation, and involution; this is coordinated by hormones and growth factors. More connective tissue and fat are available before puberty, with moderate elongation of the mammary ducts that have mammary epithelial cells invaginating into the fat pad. It should be noted that this invagination process is not dependent on hormonal action [65]. At the onset of puberty, ovarian steroid hormones accelerate the extension and branching of the mammary ducts [66]. During pregnancy, ductal branching continues. A greater branching of these ducts constitutes the lobes that are formed by alveoli. The innermost layer of the alveoli is made up of epithelial cells that differentiate and secrete milk after birth.

The MG is made up of two main structures: the parenchyma or glandular (secretory) tissue and the stroma. The secretory tissue is formed by alveoli, whose wall is covered by a simple secretory epithelium of cubic cells called lactocytes, located on a basement membrane and surrounded by an arteriovenous capillary system and myoepithelial cells, forming the outer layer of the gland, and a small population of stem cells [22]. The MG of ruminants is composed of heterogeneous tissue containing diverse populations of cells, including myoepithelial cells, fibroblasts, and adipocytes [22]. Mammary epithelial cells are the main cells present in the lactating MG and are responsible for milk synthesis. The number of mammary cells varies during lactation and is regulated by the balance between cell proliferation and apoptosis [67].

**Figure 3:**
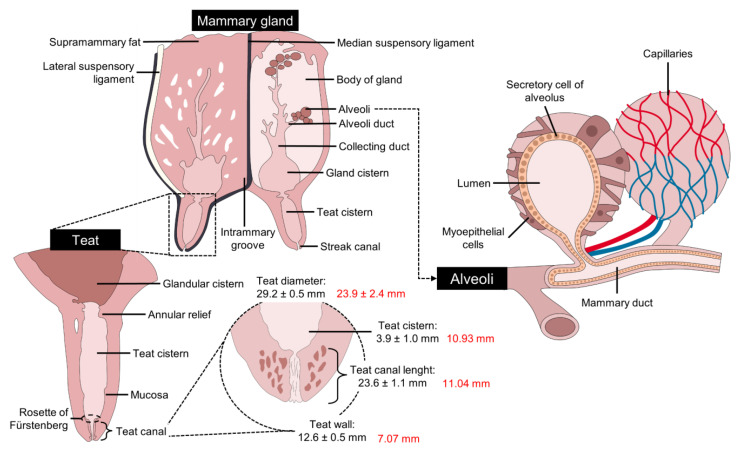
Internal structure of the mammary gland of water buffalo. Values written in red are the comparison with Holstein cattle measurements.

The alveoli are encapsulated by connective tissue (34–168 alveoli) to form the mammary lobules (*Lobuli glandulae mammariae*) [34]. These in turn join to form the lobes (*Lobi glandular mammariae*) [40]. Those that form the lobule, where milk is secreted, are emptied through small ducts, in tubules called intralobular tubules formed by bistratified cubic epithelium, which flow into a central collecting space, from which the interlobular ducts or galactophorous emerge, made of poly stratified flat and non-keratinized epithelium, releasing the milk into the so-called lactiferous ducts [68,69], The terminally differentiated mammary epithelial cells constitute the innermost layer of the alveoli. They are cuboidal cells that secrete milk proteins during lactation (Figure 3) [44,70,71].

The annular fold, the erectile venous circle, and the venous ring (Fürstenberg) are present at the junction between the gland and the cistern of the teat. The teat canal has longitudinal folds that project towards the teat duct, forming the rosette (Fürstenberg). Ozenc et al. [24] carried out comparative macroscopic examinations of the teats of cows and buffalo and the authors found that the space from the rosette (Fürstenberg) section to the teat sinus area is narrower in buffalo than in dairy cows. Furthermore, the mucosal folds observed from the teat duct to the teat sinus were more evident in buffalo than in cows. The mean length of the teat duct was 5.95 ± 0.28 and 6.37 ± 0.25 cm in the cranial and caudal teats, respectively.

The ligaments and connective tissue are essential to provide support to the udder. The quarters of the cranial glands are separated from the caudal ones by a thin septum of connective tissue not defined anatomically, while the right ones are separated from the left ones by the middle suspensory ligament. The middle suspensory ligament and the lateral ligaments emit diffuse branches towards the interior of the gland and form, together with the skin, the suspensory system of the udder [72].

## 3. Neuroendocrine Mechanisms of Milk Ejection in Ruminant Livestock

Milk ejection is the active transport of milk that is in the alveoli and transits to the cisternal compartment of the gland. It requires the contraction of the myoepithelial cells that surround the mammary alveoli and the subsequent transfer of milk through the milk duct system [13]. Milk ejection is important during milking or lactation to obtain the alveolar milk fraction, which in dairy cows can represent more than 80% of the milk stored in the alveolar compartment of the udder and only 20% in the cistern [49]. In water buffalo, it is reported that the mammary and teat cisterns contain approximately 5% of the total milk due to the small size of the cisterns of this species [50]. It should be noted that this fraction of alveolar milk is available for (machine) automatic milking or to the calf before milk ejection and requires active stimulation [49]. This ejection is induced by OXT released by the posterior lobe of pituitary gland (neurohypophysis) in response to various stimuli and a neural pathway that responds to tactile stimulation of the teat by the calf, manual massage, or the milking machine.

### 3.1. Hypothalamic–Pituitary Modulation

The milk ejection reflex is due to the nervous stimulation of mechanoreceptors located in the teats and udder. This stimulation starts with tactile stimulation of the calf or the milking machine. The transmission of nerve impulses through the udder reaches the dorsal root of the spinal cord through afferent nerve branches. Through these roots, nervous signaling reaches the hypothalamus, specifically, the supraoptic and paraventricular nuclei. Stimulation of these hypothalamic nuclei causes the release of OXT from its storage site in magnocellular neurons that extend to the neurohypophysis to be consequently released to the systemic circulation [73,74].

**Figure 4:**
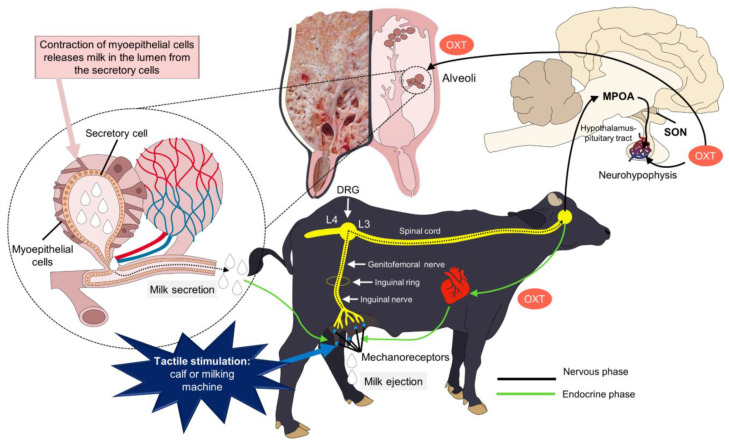
Neuroendocrinology of milk ejection. Milk ejection occurs as a response to sensorial stimulus in the mammary gland. These stimuli are transmitted through the inguinal and genitofemoral nerves to reach the dorsal root ganglion (DRG) of the spinal cord. The signaling travels through the spinothalamic tract to connect with the hypothalamus, specifically to the supraoptic nucleus (SON) and paraventricular nucleus, where oxytocin (OXT) is synthesized. Using the hypothalamic–pituitary tract, the neurons in the pituitary gland release OXT to the systemic circulation. Blood vessels in the mammary gland transport OXT to the myoepithelial cells, causing its contraction and the consequent release of milk. MPOA = medial preoptic area.

Increased plasma OXT concentrations have been reported for 10–15 min in dairy cows two minutes after the application of teat cups for milking [75]. Once OXT is transported by the systemic circulation to the udder, OXT binds to the membrane receptors located on the mammary myoepithelial cells, which surround the alveoli and small intralobular ducts. In this way, myoepithelial contraction is stimulated and causes the flattening of the alveolar lumen, forcing the milk stored in the alveoli to move into the milk ducts of the glandular and teat cistern. Finally, milk is ejected through the excretory canal of the teat (Figure 4) [49].

The ejection of milk from the alveolar cavity causes a rapid increase in pressure within the mammary cistern and, consequently, an increase in the size of the cistern cavity [49,76]. However, due to the limited space of the bovine mammary cistern, not all alveolar milk can be expelled if it is not simultaneously extracted from the udder [77]. On the other hand, an empty mammary cistern during milking can cause impaired milk extraction. At the end of milking or suckling, complete milk secretion must be achieved to maintain a high level of milk synthesis and secretion during lactation and to reduce the risk of infection by the presence of residual milk [49].

### 3.2. Milk Ejection

The MG physiology of buffalo differs slightly from that of cattle [50]. Continuous and complete emptying of the MG depends directly on elevated OXT concentrations [78]. However, morphological differences are related to differences in buffalo’s milk letdown [79]. In buffalo, milking is possible only after the milk has been expelled from the teat cistern. Therefore, no milk is available in the teat cistern after teat cannulation. The total area of the glandular cistern and the fraction of the cisternal milk in female buffalo is smaller than in dairy cows, sheep, and goats since the cisternal fraction represents an average of 5% of the total milk a trait that decreases during lactation and increases with age [78].

**Figure 5:**
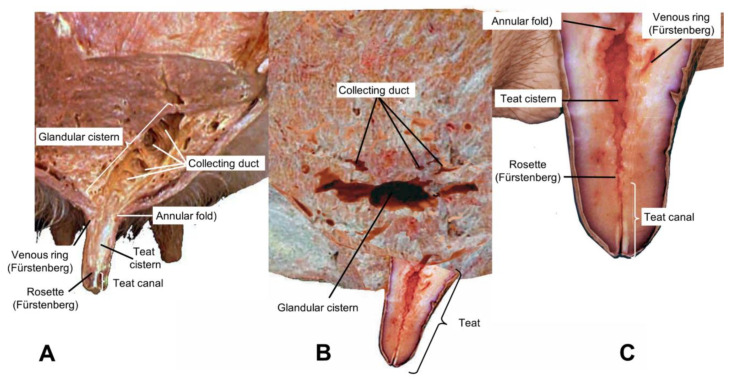
Comparative anatomy of the mammary glands in lactating cows and buffalo: (**A**)**:** the sagittal section of the glandular mammary of the adult lactating cow; (**B**)**:** the sagittal section of the glandular mammary of the adult lactating buffalo; and (**C**)**:** the sagittal section of the teat of the adult lactating buffalo.

The small volume of the buffalo’s glandular cistern suggests that longer stimulation of the udder is necessary before cup placement to ensure proper OXT release and proper milk letdown (Figure 5); however, other elements influence the requirement of more stimulation such as animals in later lactation with lower milk production. Additionally, buffalo teats are longer and thicker and have longer ducts than those of dairy cows [50].

This is one of the reasons why buffalo milking is considered slow, due to their slow milk expulsion reflex and hard teat sphincter muscle [12]. Due to this characteristic, the delay time between teat stimulation and milk letdown requires more time compared to cows, lasting between 2 and 3 min [47]. However, milk ejection can last up to 10 min influenced by the release of OXT.

Moreover, genetic selection for milkability traits in dairy cows might influence the shorter time for milk letdown. For example, Visscher and Goddard [80] found that heritability for milking speed in Holstein and Jersey cattle ranged from 0.18 to 0.29, results that were similar to those reported by Wethal and Herinstad [81] in Holstein-Friesian and Jersey cattle for average flow rate or milk kg/minute (0.48 and 0.27, respectively). Genomic-wide studies have also compared milk production traits between dairy cattle and water buffalo. Liu et al. [82] found that genomic regions affecting milk fat and protein percentage (BTA3) and regions influencing total milk, fat, and protein yield (RGS22 and VPS13B) were present in both species and that genomic estimated breeding values of milk trait range between 0.06–0.22 in Italian Mediterranean buffalo. In Murrah buffalo, genomic studies have found moderate heritability estimates for milk yield (0.35 ± 0.02), fat yield (0.22 ± 0.03), and protein yield (0.42 ± 0.03) [83]. Similar results were found in another study using the same breed of buffalo, in whom a heritability for milk yield of 0.31 ± 0.11 was reported [84], while heritability coefficients of 0.17 for milk yield were found in cross-breed buffalo [85]. Nonetheless, further studies are required to determine the heritability of milking parameters in water buffalo as they have been established in *Bos* cattle [51].

On the other hand, intramammary pressure during milking is greater in buffalo. An increase has been recorded at the beginning of milking, and it becomes even higher during peak flow, contributing to milk ejection. Although intramammary pressure varies between individuals and milking, its level is not indicative of high milk production [12]. In addition, the milk ejection reflex has a significant effect on milk quality. During automatic milking, the concentration of milk fat increased over time since the milk extracted at the end of a single milking, corresponding to alveolar milk, is 2.5 to 5 times richer in milk fat compared to cisternal milk [75].

Continuous milk ejection depends on high concentrations of OXT [75]. The record of basal levels in OXT concentration has been 4.8 to 6.7 ng/L; at maximum concentrations of 90 ng/L, but during teat stimulation, milking, and feeding during milking, values of approximately 30 ng/L have been recorded. This physiological action is linked to milking time and continuous stimuli of the afferent nerves [12]. On the other hand, it is well known that the administration of OXT before milking in large buffalo herds is frequently used to achieve milk letdown [50]. Therefore, milk ejection requires the presence of circulating OXT and proper stimulation for its release.

## 4. Stimulus That Promotes Milk Ejection in Ruminant Livestock

The neuroendocrine reflex for milk letdown occurs in response to the presence of the calf, suckling, manual stimulation (cleaning/disinfection), feeding during milking, and exogenous administration of OXT [13,47,86,87]. Due to the low proportion of cisternal milk in buffalo, stimulation of the MG is required before milking. This action will allow milk to be collected from the alveolar compartment in response to the activation of milk letdown. Therefore, the milking units (cups or teat cups) must be placed after the start of the milk ejection response [50]. Some authors mention that a prestimulation of 60 s improves milking in Italian Mediterranean buffalo [88], while other studies indicate that the optimal duration of prestimulation is 1–2 min [47]. However, the latency and time for prestimulation depend on the filling of the udder [89].

The stimulation required before milking can be influenced by the stimulation technique or by breed characteristics [90,91], the stage of lactation [92], the relative degree of udder filling, and the milking interval [93]. However, most studies investigating premilking stimulation have focused on describing the effects of premilking stimulation on milk production to improve parlor efficiency and profitability of milking.

### 4.1. Visual–Tactile Stimulation for Water Buffalo

For more than 40 years, it has been observed that the presence of the suckling calf during milking enhances maternal secretion of oxytocin. In developing countries, such as India and Pakistan, manual milking is common, and the technique is based on the calf’s presence for a limited time (approximately 2 min). However, other authors report that the presence of the calf with its mother reduces milk production [94,95], as observed in where milking using the calf as stimulation resulted in a milk yield of 2.16 kg, while buffalo receiving oxytocin administration and manual stimulation had an average milk yield of 2.36 and 2.17 kg, respectively [96]. In contrast, when calves suck and stimulate the udder, milk secretion increases [19,21,50,97,98], where suckled buffalo had a daily milk yield of 10.2 ± 0.2 kg while non-suckled females had an average production of 7.8 ± 00.2 kg [99]

Tactile stimulation (manual or mechanical) of the MG causes efficient milk ejection [100,101]. Whether in conventional or automatic milking, preparing the teats before milking consists of ensuring the cleaning and disinfection of the teats as well as the complete expulsion of milk [101].

**Figure 6:**
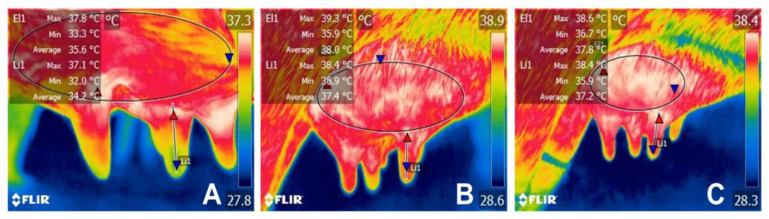
Surface thermal response of the udder of Murrah buffalo before, during, and after suckling. (**A**) Before suckling, the maximum temperature of the udder and the teat is 37.8 °C and 37.1 °C, respectively. (**B**) During colostrum intake by the calf, the maximum temperature of the udder and the teat reaches 39.3 °C and 38.4 °C, increasing an average of up to 1.5 °C when compared to the previous thermal image. (**C**) After suckling, a decrease in the maximum temperature of the udder (38.6 °C) is observed when compared to the B image. However, in comparison to A, both temperatures stay above basal temperature by up to 1.4 °C. Red triangle: maximum temperature; blue triangle: minimum temperature. Radiometric images were obtained using a T1020 FLIR thermal camera. Image resolution: 1024 × 768; up to 3.1 MP with UltraMax. FLIR Systems, Inc. Wilsonville, OR, USA.

Thomas [50] reports that buffalo manually stimulated one minute before milking secrete a slightly greater amount of OXT than buffalo without prior stimulation (18.04 ± 5.85 ng/L vs. 6.31 ± 5.85 ng/L, respectively) and that the secretion of OXT is even greater if manual stimulation is combined with the supply of feed (or concentrates) during milking (47.86 ± 5.85 ng/L). Furthermore, stimulation by the calf also increases the blood flow to the udder, improving the neurophysiological response required for milk synthesis and ejection. This was observed through infrared thermography in a preliminary study made by the present authors where the surface temperature of the udder and teats was evaluated in Murrah buffalo before, during, and after suckling bouts to provide colostrum to the calves (Figure 6). The increases in temperature during suckling of the calf might be related to the tactile stimulation to the greater vascularization in the udder and the presence of hormones such as estrogen after calving.

### 4.2. Water Buffalo’s Milking Routine

The milking routine of animals highly influences their welfare, since some interaction with stockpeople and the environment might be regarded as stressful for the animals [102,103,104]. Buffalo are more sensitive to stress stimuli than cattle (*Bos taurus* and *Bos indicus*) [50]. Inadequate animal handling such as shouting or hitting, as well as deficient maintenance of milking machines can be regarded as stressful for buffalo [18,105,106,107,108].

If animals are under acute stress, adrenaline is released, reducing the supply of OXT necessary for milk ejection. Adrenaline release causes vasoconstriction of mammary blood vessels, limiting OXT action [96,109,110]. Moreover, this inhibits the contraction of the myoepithelial cells in the alveoli [111,112]. In buffalo, it has been reported that even small changes in the milking routine can make the animal uncomfortable and disturb milk ejection [113]. In a study by Polikarpus et al. [111] on buffalo behavior during milking, a strong consistency in their milking routine was observed, expressing a preference for the order of entry to the parlor and location options. According to Mellor et al. [114], positive stimulation can be provided to dairy animals by giving animals an adequate space to move, using an appropriate substrate and a well-drained soil, fresh air that disperses pollutants, and overall satisfactory hygiene. Moreover, the availability of shelter and shade, as well as controlling the amount of noise and light might enhance the welfare of animals during milking.

### 4.3. Negative Stimuli: Inhibition of Milk Ejection

On the other hand, some negative stimuli or factors that might be present in dairy systems can have detrimental effects on animals [115,116,117,118]. Buffalo are sensitive to changes in their environment, even the most subtle ones. Negative stimuli are related to the situation in which the animals find themselves, specifically to the experiences generated by the brain processing of sensory inputs that originate mainly from the outside. The physical environmental conditions that produce negative stimuli frequently correspond to confinement, and overcrowding; inadequate substrate and wet/dirty soil; air pollutants: NH_3_, CO_2_, dust, and smoke; unpleasant odors, thermal extremes; loud and/or unpleasant noise; inappropriate light intensity; monotony: environmental, physical, and lighting; and unpredictable events and physical limits for rest and sleep [114].

The level of noise inside the milking parlor, whether continuous or intermittent, coming from a variety of sources in the environment can be considered a stressor and can trigger harmful stress responses [116,119]. Although studies evaluating the effect of noise on water buffalo’s performance are limited, in dairy farming, a study evaluated the effect of noise on the behavioral response of animals, finding that heifers exposed to noise from commercial milking facilities show escape behavior, which is considered a fear response [115]. For example, detrimental effects have been found in dairy cows exposed to noise levels above 70 dB, and is recommended to establish protocols so the noise level does not exceeds 65–70 dB [120]. Cwynar and Kolacz [121] reported that noises of 75, 85, and 95 dB with frequencies of 2 kHz reduce the animals’ appetite. Other authors documented a reduction in milk production in cows exposed twice a day to noise levels between 80 and 100 dB for more than one hour [122]. This has been associated not only with tachypnea and tachycardia but also with altered productive performance [122,123].

Brouček [116] highlights that the available evidence suggests an alteration in carbohydrate metabolism in ruminants exposed to a variety of noises produced by an industrial engine or human vocalizations. The activation of the sympathetic-adrenal system can totally or partially inhibit the milk ejection reflex [96]. Said withdrawal could be of central or peripheral origin. The first includes interferences in the release of OXT by the neurohypophysis, while peripheral alterations occur when there are disorders in the expulsion of milk from the MG. These disorders involve different physiological mechanisms that prevent the access of OXT to the MG by blocking OXT receptors or hinder milk letdown from the alveolar compartment to the cisterns during milking as a consequence of a high concentration of endogenous OXT [124]. Moreover, adrenaline causes vasoconstriction of the blood vessels and capillaries of the MG, reducing the supply of OXT and inhibiting the contraction of the myoepithelial cells of the alveoli [125].

**Figure 7:**
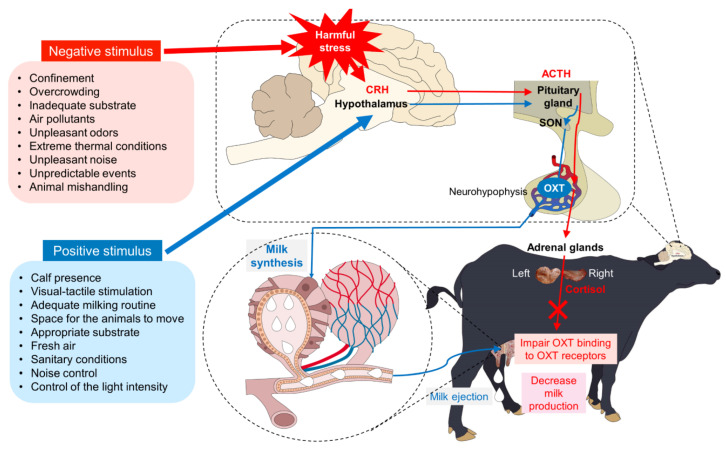
Positive and negative stimulus that influence milk synthesis and ejection. CRH: Corticotropin-releasing hormone; ACTH: adrenocorticotropic hormone; SON: supraoptic nucleus; OXT: oxytocin.

Continuous exposure to harmful stress causes a decrease in milk production. Some of the inhibitory causes of milk ejection are suckling alien calves, milking primiparous females immediately after parturition or recently weaning, changes in stockpeople (milker), application of inadequate routines, erroneous techniques, and milking equipment in skimpy condition [78,126]. Furthermore, human beings also influence the external circumstances of animals, and their interactive behavior towards animals has the potential to cause positive effects that improve their welfare or, conversely, negative effects that compromise their mental state [114]. Figure 7 summarizes some of the positive and negative stimuli that intervene in the milk ejection mechanism.

## 5. Conclusions

The udders of water buffalo and dairy cattle have significant anatomical differences that can influence the milking process. For example, buffalo’s udders and suspensory ligament systems are less developed than those of cows. This implies that buffalo produce less milk than dairy cattle.

In buffalo, 95% of milk is stored in the secretory tissue even after a milking interval of 10 to 12 h. Due to the low proportion of cisternal milk in buffalo, the stimulation of udder before milking (between 2 to 3 min before milking) is essential. To enhance milking time, a reduction in stressors such as noise, feeding during milking, and an acceptable environment with good lighting are recommended for buffalo. Preventing stress in animals during milking or lactation prevents the release of catecholamines that alter OXT function on mammary OXT receptors. The correct implementation of these measures promotes optimal environmental conditions that allow maximum milk synthesis and ejection. Negative factors like confinement, overcrowding, and high noise levels can harm buffalo welfare, leading to increased harmful stress and decreased milk production.

## Figures and Tables

**Table 1:** Morphologic measurements of the udder and teats of different breeds of water buffalo.

	Murrah	Murrah	Murrah	Egyptian	Mediterranean Italian	Nili Ravi	Jaffarabadi
Cisternal area (cm2)	47.56 ± 11.10	-	-	-	-	-	-
Cisternal fraction (%)	4.9 ± 0.1	-	-	-	-	-	-
Fore teat length (cm)	9.02 ± 0.45–10.42 ± 0.47	7.37 ± 0.14–7.53 ± 0.15	5.42 ± 0.02–5.60 ± 0.02	6	7.01 ± 0.17	9.6 ± 1.2	7.69 ± 0.10–7.79 ± 0.11
Hind teat length (cm)	11.80 ± 0.47–13.15 ± 0.41	8.23 ± 0.18–8.12 ± 0.16	5.74 ± 0.03–5.96 ± 0.02	6.3	8.33 ± 0.22	-	8.63 ± 0.16–8.67 ± 0.16
Fore teat circumference (cm)	10.31 ± 0.38–11.90 ± 0.45	-	-	-	-	-	-
Hind teat circumference (cm)	11.68 ± 0.36–13.29 ± 0.42	-	-	-	-	-	-
Teat diameter (cm)	-	2.76 ± 0.02	-	2.2–2.3	3.28 ± 0.05	4.08 ± 0.66	3.17 ± 0.03
Teat canal length (cm)	-	-	-	1.3	2.63 ± 0.09–2.78 ± 0.11	-	-
Teat wall (cm)	-	-	-	-	2.44 ± 0.44	-	-
Udder length (cm)	-	54.2 ± 0.34	47.44 ± 0.37–51.55 ± 0.90	-	-	64.2 ± 7.3	65.75 ± 0.52
Udder width (cm)	-	50.6 ± 0.36	41.81 ± 1.18–46.15 ± 0.94	16–29.2	-	29.1 ± 4.1	51.19 ± 0.26
Udder depth (cm)	-	15.5 ± 0.10	11.67 ± 0.05–12.30 ± 0.20	10–30	-	10.8 ± 1.6	18.16 ± 0.22
Udder circumference(cm)	-	-	-	75–85.4	-	-	-
Reference	[52]	[54]	[62]	[63]	[27]	[26]	[64]

-: not assessed in the study.

## Data Availability

Data sharing not applicable.
